# Announcements and Corrections

**Published:** 1914-07

**Authors:** 


### ANNOUNCEMENTS AND CORRECTIONS

---

As a matter of convenience, it has been decided to establish a department of this nature, at the end of each issue, as necessary.

Paul B. Hoeber asks us to correct the price of the two booklets by Sir Wm. Osler as stated in Book Reviews, page 713, June issue. The price is $1.00, not $1.50.

The full report of the Commencement Exercises and Alumni meeting of the University of Buffalo, which we expected to publish in this issue, has been delayed, by various demands on the time of the Committee. As it is desired to present the report in full, it has been deemed wise not to anticipate it by the publication of an incomplete report. It is expected that the Harrington Lectures by Prof. Ludwig Pick will also be published in this Journal, probably beginning in the September issue.

Original articles for the August and September issues are already arranged and partly printed. Intending contributors are requested, in order to secure prompt publication, to send in articles as soon as possible. Brief articles, requiring early publication to secure priority or for other reasons, can usually be printed in the next month's issue, if received by the fifth of any month.

Contributors should bear in mind that it is contrary to the policy of this Journal to publish ''addresses'' unless these are, at the same time, papers on practical medical subjects. Brief notes on strictly historic matters and discussions of live professional issues, not too polemic and not too long, are not included in this exclusion.

We are going to ask our subscribers to increase their subscription to this extent: We would like each subscriber, on the average, to send in one personal or other news item, every year; to read the advertising section thoroughly at least once every three months; to turn over one copy of the Journal to a non-subscriber, once a year. These may seem like trivial requests and it may be asked why, if we are asking favors at all, we do not ask something more. Because, by actual calculation, this amount of co-operation is all that is necessary to put the Journal on the highest level of efficiency and excelience for the purpose which it seeks to fulfill.

